# Effects of a long-acting trace mineral rumen bolus upon range cow productivity

**DOI:** 10.1093/tas/txaa232

**Published:** 2020-12-24

**Authors:** James E Sprinkle, David W Schafer, S Peder Cuneo, Douglas R Tolleson, R Mark Enns

**Affiliations:** 1 Department of Animal and Veterinary Sciences, University of Idaho, Moscow, ID; 2 University of Idaho Nancy M. Cummings Research, Extension and Education Center, Carmen, ID; 3 Agricultural Research, Development, and Education Center, Colorado State University, Fort Collins, CO; 4 School of Animal and Comparative Biomedical Sciences, University of Arizona, Tucson, AZ; 5 Texas A & M AgriLife Research, Sonora, TX; 6 Department of Animal Sciences, College of Agricultural Sciences, Fort Collins, CO

**Keywords:** beef cattle, calving interval, copper, minerals, range, selenium

## Abstract

The objectives were to determine if strategic supplementation of range cows in central Arizona with either two or four long acting (6 mo) trace mineral rumen boluses containing Cu, Se, and Co would: 1) decrease yearly calving interval; 2) increase cow body condition, milk production, or calf adjusted weaning weights; and 3) to see if any of the above traits varied by cow breed. There were 194 Hereford (H) and 132 Composite (CGC; 50% Red Angus, 25% Tarentaise, 25% Charolais) control cows, 173 H and 125 CGC 1X treated (2 boluses in late winter) cows, and 183 H and 117 CGC 2X treated (2 boluses in autumn and 2 in late winter) cows used over the 4-yr period. Cows were weighed and scored for body condition (1–9, 9 = fattest) in February, May, and September of each year. Milk production was determined by weigh-suckle-weigh on a subset of cows (*n* = 169) at an average of 50 d lactation. The outcomes were analyzed using a restricted maximum likelihood-based mixed-effects model that included the categorical, fixed effects of breed, bolus, and year with the interactions of breed × bolus, and breed × year. For adjusted weaning wt (WW), year × bolus was added. The random effect of cow was also included. Calving interval had only the breed × bolus interaction added to the main effects. Age of dam was added as a covariate to all models. Milk production used the same model as calving interval with the added covariate of postpartum interval. Cow body condition score and calf adjusted weaning weights differed by breed and treatment (*P* < 0.05) with WW being greater (*P* < 0.05) for calves from 2X cows than for control calves. Milk production differed by year (*P <* 0.0001) but did not differ by either breed or treatment (*P* > 0.05). Calving interval was 389 ± 2.7, 382 ± 3.2, and 378 ± 3.2 d for control, 1X, and 2X treatments, respectively and calving interval declined (*P* < 0.05) from the control to the 2X treatment group. Strategic supplementation via a long-acting trace mineral bolus was successful in decreasing calving interval and increasing calf-weaning weights from cattle grazed in an extensive rangeland environment.

## INTRODUCTION

Rangelands throughout the Western United States are often deficient in minerals needed for optimal livestock production (Corah and Dargatz, 1996; Mathis and Sawyer, 2004; Sprinkle et al., 2006; Sprinkle et al., 2018). Chief among reported trace mineral deficiencies in rangeland forage include Cu, Se, Co, and Zn (Corah and Dargatz, 1996; Sprinkle et al., 2018). Clinical signs of malnutrition for these trace minerals include abnormal hair coloration, joint popping and lameness, chronic scours, weak calf syndrome, white muscle disease, retained placentas, and foot rot (Maas, 1983; Corah and Ives, 1991; Puls, 1994; Radostits et al., 1994; Carpenter et al., 1996). Subclinical signs of trace mineral malnutrition include impaired fertility, reduced growth, and reduced immunity (Maas, 1983; Corah and Ives, 1991; Corah and Dargatz, 1996; Ward and Spears, 1997; Galyean et al., 1999; Greene, 2000). All of these nutritional deficiencies in Western range cows impacts overall profitability and ranch sustainability.

A long acting (6 mo) rumen trace mineral bolus containing Cu, Se, and Co has been developed in the United Kingdom (Cosecure, Bimeda UK, Anglesey, Wales) and has shown promise for helping alleviate trace mineral deficiencies (Buckley et al., 1987; Givens et al., 1988; Sprinkle et al., 2006). One advantage of the long-acting rumen boluses is the capability to provide a trace mineral supplement to livestock grazing on expansive rugged topography rangelands that are inaccessible by motor vehicles. In a previous study in central Arizona, Sprinkle et al. (2006) found that use of the Cosecure bolus caused increased weight loss (*P* = 0.02) from late gestation to early lactation, but milk production was not determined in that study. In addition, the same study failed to report the effect of the long acting rumen boluses upon yearly calving interval.

The objectives of this study were to examine the effects of the Cosecure boluses supplemented either one or twice per year upon body condition score (**BCS**), body weights, yearly calving interval, milk production and calf weaning weights, and to see if any of the above traits were subject to a breed by treatment interaction.

## MATERIALS AND METHODS

Care, handling, and sampling of the animals were approved by the University of Arizona Institutional Animal Care and Use Committee (Protocol No. 06-004).

### Grazing Environment

The study site for this experiment was at the 32,161 ha V-V Ranch operated by the University of Arizona and located near Camp Verde, Arizona. Slightly more than 16 ha is privately owned and the remainder of the ranch is a public lands grazing permit (Walker Basin Allotment) administered by the U.S. Forest Service. The ranch is extensive in nature with much of the ranch only being accessed by primitive dirt roads, off-road vehicles, and horseback. The ranch ranges in elevation from approximately 975 m (low desert shrub range type) to 2,195 m (Ponderosa pine montane range type). A transitional pinyon–juniper range type between the upper and lower elevation ranges averages around 1,600 m. Dominant perennial herbaceous grasses at the low desert site included sideoats grama (*Bouteloua curtipendula* [Michx.] Torr.), slim tridens (*Tridens muticus* [Torr.] Nash), sand dropseed (*Sporobolus cryptandrus* [Torr.] A. Gray), black grama (*Bouteloua eriopoda* [Torr.] Torr.), and threeawn (*Aristida* spp). Dominant perennial grasses at mid-elevation pinyon–juniper site included sideoats grama, blue grama (*Bouteloua gracilis* [Willd. Ex Kunth] Lag. Ex Griffiths), vine mesquite (*Panicum obtusum* [Kunth]), spike muhly (*Muhlenbergia wrightii* Vasey ex Coult.), and western wheatgrass (*Elymus smithii* [Rydb.] Gould), and the dominant half-shrub shrubby buckwheat (*Eriogonum wrightii*). The dominant herbaceous species that characterized the upper elevation Ponderosa pine sites included bottlebrush squirreltail (*Elymus elymoides* [Raf.] Swezey ssp. Elymoides), Kentucky bluegrass (*Poa pratensis* L.), blue grama, spike muhly, western wheatgrass, and elk sedge (*Carex geyeri*).

The majority of the ranch (mid- and upper elevation) contained soils derived from basalt parent material and the lower elevation areas of the ranch contained some basaltic soils but with limestone outcroppings and alluvial and colluvial deposits of sandstone, limestone, and basalt dominating.

Average yearly precipitation ranges from 32 cm at the lower elevations to 65 cm at the upper elevations. However, annual precipitation during the course of this trial was quite variable at the Happy Jack (**HJ**, upper elevation) and Montezuma Wells (**MW**, lower elevation) weather stations (Table 1), with more winter moisture in 2005 before the study commenced; substantially less annual precipitation in 2006, especially in winter and early spring; about the same and slightly less annual precipitation in 2007 at MW (31 cm) and HJ (60 cm), respectively with less early fall rainfall at MW and less winter and early spring moisture at HJ. A wet El Niño winter occurred in 2008 at all elevations with less early spring and early fall moisture at HJ and less late summer moisture at MW; and substantially less precipitation at all elevations in 2009, particularly during the midsummer and early fall growing season for warm season grasses.

**Table 1.** Precipitation totals for the V-V Ranch, cm

	Upper elevation^*^												
Year	Jan.	Feb.	Mar.	Apr.	May	June	Jul.	Aug.	Sep.	Oct.	Nov.	Dec.	Total
Average	7.19	7.06	7.59	3.30	2.18	0.86	6.76	7.77	5.74	4.45	4.80	6.81	64.85
2006	1.19^c^	0.00	6.30^a^	0.18	0.48	1.98^d^	5.41	9.53	3.73	2.90	0.46	1.02^c^	33.17
2007	5.05^a^	6.02^c^	3.10	1.12	0.38^a^	0.00^a^	11.07	8.53^e^	6.71	0.53	0.30	17.50^c^	60.33
2008	21.84^c^	12.75^j^	0.23^a^	0.38	2.06	0.03	10.01^k^	10.26	1.65	0.58	5.16^k^	15.44^g^	37.03^d^
2009	3.91^e^	9.55^c^	0.00^d^	1.19^h^	7.57^b^	0.74^b^	2.08^a^	4.14	5.66^c^	0.20	1.07^h^	9.75	43.61^b^
	Lower elevation^†^												
Year	Jan.	Feb.	Mar.	Apr.	May	June	Jul.	Aug.	Sep.	Oct.	Nov.	Dec.	Total
Average	2.87	2.87	2.84	1.63	0.86	0.66	3.91	5.28	3.81	2.54	2.13	3.25	31.90
2006	0.43	0.00	1.50	0.76	0.00	0.30	7.75	4.47	6.05	2.46	0.00	1.09	24.82
2007	1.93	2.67	2.64	0.13	0.23	0.00	6.76	4.75	2.87	0.05	0.56	8.23	30.81
2008	6.07	2.67	0.00	0.00	2.79	0.00	3.99	4.17	2.21	0.00	3.15	5.59^a^	30.63
2009	3.02	2.57	0.74	0.64	4.70	0.33	0.64	1.27	2.95	0.00	0.41	3.48	20.73

*Upper elevation (2,280 m) average precipitation data (1970–2019) and precipitation data for 2006–2009 were obtained from the Happy Jack Arizona Ranger Station, available at http://www.wrcc.dri.edu/. ^a^ = 1 d missing; ^b^ = 2 d missing; ^c^ = 3 d missing, etc.

^†^Lower elevation (969 m) average precipitation data (1939–2019) and precipitation data for 2006–2009 were obtained from the Montezuma Castle National Monument, available at http://www.wrcc.dri.edu/. ^a^ = 1 d missing; ^b^ = 2 d missing; ^c^ = 3 d missing, etc.

Cattle grazed through 37 upland pastures from low and mid-elevation pastures in winter and spring to mid-elevation and upper elevation in summer and fall in a modified holistic management-grazing plan (The Savory Center, Albuquerque, NM) as described further by Tolleson and Schafer (2014). Cattle were typically moved every 10–20 d.

### Forage Sampling

Forage was sampled by hand clipping from the above range sites which cattle were grazing at the time cattle received supplemental trace mineral boluses (February or March for desert shrub and pinyon–juniper sites and September for Ponderosa pine sites). All dominant forage species listed above were sampled yearly from 2006 to 2009. The grass samples were clipped to ground level by species and shrubby buckwheat had the current year’s leaders clipped. Plant samples were approximately 150 g per species from plants distributed randomly over the sampling area. These 13 different forage species were sampled for nutritional adequacy of Ca, P, Mg, K, Na, Cu, Se, Co, Mn, and Zn for each year and for the concentrations of S, Mo, and Fe to see if antagonistic interactions existed. Prior to mineral analysis, forage samples were air dried at ambient temperatures, then shipped to the Oscar E. Olson Biochemistry Analytical Services Laboratory in Brookings, SD where they were ground to pass through a 1 mm screen using a Tecator Cyclotec (Foss, Hilleroed, Denmark) cyclone pulverizing mill (AOAC, 2005). Samples were then mixed and moisture determined (on a subsample) at 105°C for 3 h in a mechanical convection oven (Method 2.1.4, NFTA, 2006), then analyzed fluorometrically for Se following digestion in percholoric and nitric acids and reduction with 0.1 M HCl and complexation with diaminonapthalene (Olson et al., 1975; Koh and Benson, 1983; Palmer and Thiex, 1997) as reported in AOAC (Official Method 996.16, AOAC, 2005). Following these analyses, the samples were shipped to Dairy One Lab in Ithaca, NY and analyzed for Ca, P, Mg, K, Na, Fe, Zn, Cu, Mn, Mo, Co, and S using inductively coupled, plasma emission spectroscopy as described by Sirois et al. (1991).

### Animals

The trial commenced in October 2005 and concluded in September 2009. Treatment and control cattle were randomly allocated at the onset and remained in each treatment group throughout the 4-yr trial. There were 194 Hereford (**H**) and 132 Composite (**CGC**; 50% Red Angus, 25% Tarentaise, 25% Charolais) control cows, 173 H and 125 CGC 1X treated (2 boluses in late winter) cows, and 183 H and 117 CGC 2X treated (2 boluses in autumn and 2 in late winter) cows used over the 4-yr period. Cows ranged in age from 2 to 12 and 2 to 10 yr for H and CGC, respectively.

In September or October and February or March of each year, cows in the 2X treatment groups were orally dosed with 2, 100 gram Cosecure (Bimeda UK, Anglesey, Wales) boluses consisting of 0.30% (wt/wt) selenium as sodium selenate, 13.4% (wt/wt) copper, and 0.5% (wt/wt) cobalt. The 1X treatment group only received boluses in February or March. According to company literature validated with rumen fistulated cattle on a silage and concentrate ration, boluses dissolved in 175 days and released 156, 5.9, and 3.4 mg/d of Cu, Co, and Se, respectively.

A subset of mature cattle (5–10-yr-old) from each treatment group were sampled for milk production near expected time for peak lactation (50 d) in 2006, 2008, and 2009 using the weigh-suckle-weigh technique described by Williams et al. (1979). There were 26 CGC Control, 31 CGC 1X, 28 CGC 2X, 28 H Control, 26 H 1X, and 30 H 2X cows used over all 3 yr of milk production data collection. Calves were removed from cows the night before determining milk production with an average calf separation time of 8.45 h in 2006 for eight different groups, 9.15 h in 2008 for six different groups, and 12.88 h in 2009 for seven different groups. Cattle were not sampled in 2007 due to a lack of an adequate sample size of cows available at peak lactation.

Cattle remained in a common herd as they moved through the 37 upland pastures. Cattle did not receive any type of oral trace mineral supplement for the 4 yr of the trial except for free choice white iodized salt blocks and the incidental minerals contained in protein blocks used from April to June in 2006 and from March to May 15 in 2008 (27% crude protein, 17 ppm Cu, 0.301 ppm Se, Eagle Milling Co., Inc., Casa Grande, AZ). At an average daily protein supplement intake of 0.916 kg in 2006 and 0.395 kg in 2008, it was estimated that cattle received an additional 16 mg/d of Cu and 0.28 mg/d Se in 2006 and 7 mg/d of Cu and 0.12 mg/d of Se in 2008.

The majority (74%) of calves born in this trial were sired by Hereford bulls via artificial insemination or pasture exposure. Other sire breeds represented were Waguli (13%), Tuli (4%), Wagyu (3%), Red Angus (3%), Angus (2%), and miscellaneous (1%). Breeding seasons extended from May 20 to September 6 in 2005, May 18 to August 30 in 2006, May 15 to September 20 in 2007, May 23 to September 4 in 2008, and from May 22 to August 5 in 2009. Cows were artificially inseminated once following estrus synchronization using Easi-Breed CIDRs (Pharmacia & Upjohn Co., Kalamazoo, MI), then pasture exposed to bulls.

In September to October and February to March, pregnancy was determined by rectal palpation. Cattle were weighed and scored for BCS (1–9; 9 = fattest; same trained observer) four times per year in February or March, May or June, and September or October. Birth and weaning weights were collected on all calves. The majority of the calves were weaned in October at approximately 182 d of age and weaning weights were adjusted to 205 days of age and for age of dam according to BIF (2018) guidelines.

### Statistical Analyses

Cattle production data were analyzed using a restricted maximum likelihood-based mixed effects model appropriate for repeated measures (SAS Inst., Inc., Cary, NC) with the categorical, fixed effects of breed, bolus treatment, and year with the interactions of breed × bolus treatment, and breed × year. For adjusted weaning weight, year × bolus treatment was added to the model. Cow within breed by bolus was included as a random effect. Calving interval had only the breed × bolus interaction added. Age of dam was added as a covariate to all models. Milk production used the same model as calving interval with the added covariate of postpartum interval. The denominator degrees of freedom for treatment *F*-statistics were approximated using the Kenward-Roger’s method. For all models except calving interval, a heterogeneous autoregressive structure was used as a covariance structure to model the relationships between repeated observations. In order for calving interval to properly converge with this iterative methodology, a simplified compound symmetry covariance structure was used. Forage data were analyzed by mixed model procedures with forage species, year, and pasture as fixed effects and plant species within (pasture) as the repeated measure. The denominator degrees of freedom for forage were approximated using the Satterthite method and the compound symmetry covariance structure was used for convergence. To assist with the convergence of the forage mixed analysis without encountering a nonpositive Hessian matrix, starting parameter values were added to the analysis program code for all the forage mineral analyses except for S, Fe, Cu, and Mn. Treatment means for all statistical models were separated using the PDIFF function in SAS (SAS Inst., Inc., Cary, NC).

## RESULTS AND DISCUSSION

### Overall Forage Mineral Concentrations

**Figure 1.**
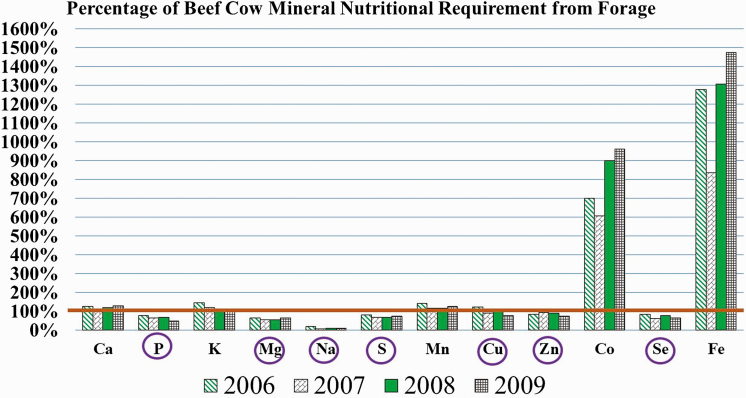
Yearly average mineral content of native range forage in central Arizona expressed as a percentage of daily requirements. Nutrient requirements are based upon NASEM, 2016, Nutrient Requirements of Beef Cattle. The Ca and P requirements are based upon a 480 kg cow with 6 kg peak milk production, 34 kg calf birth weight, and 195 kg adjusted calf weaning weight. Circled minerals indicate years in which forages alone failed to meet nutritional requirements. The antagonistic mineral Mo did not have a sufficient amount to hinder Cu absorption, only averaging 0.60 ppm over all years of the study.

Concentrations of macrominerals in the available forage were adequate for Ca and K while concentrations of P, Mg, Na, and S were considered deficient (NASEM, 2016) among all years of the study (Figure 1). Among microminerals, available forage was deficient (NASEM, 2016) among all years of the study for Se and Zn and for 2 of the 4 yr for Cu (Figure 1). Cobalt and Fe were greatly above (NASEM, 2016) nutritional requirements in the available forage (Figure 1) and Fe was present at levels in the forage sufficient to hinder Cu absorption (>400 ppm; Corah and Dargatz, 1996). The micromineral Mo is considered to be antagonistic for Cu absorption if present in forage at levels greater than 2 ppm (NASEM, 2016), particularly when combined with higher levels of sulfur (>0.2–0.3%; Mortimer et al., 1999; Ivancic and Weiss, 2001). These antagonistic minerals of Mo and S contribute to the formation of insoluble thiomolybdate complexes in the rumen that hamper Cu absorption by livestock (Gould and Kendall, 2011). The forage tested in this study did not contain high levels of either S or Mo; S averaged 0.12% over all years of study and Mo averaged 0.60 ppm. Therefore, the main concern for antagonistic mineral interactions on this ranch was for high levels of Fe (Sprinkle et al., 2006), which is not uncommon for native range forages (Mathis and Sawyer, 2004). High Fe levels been implicated for the disruption of Cu absorption in the animal as well as interfering with the availability of Se. The plant uptake of Se is hampered in clay soils (Johnsson, 1991; Sprinkle et al., 2018) due to prevalent Fe_2_O_3_, which binds to Se. The majority of soils on this ranch are derived from basaltic parent material, which predominantly weather to a heavier clay soil.

Copper, Se, Zn, and Co have often been identified as trace minerals of concern for nutritional deficiencies in native range grasses in the Western states (Corah and Dargatz, 1996; Sprinkle et al., 2006; Sprinkle et al., 2018). We only provided supplemental trace minerals through the use of the Cosecure boluses (Cu, Se, and Co) and by incidental amounts present in the protein supplement fed in 2006 and 2008. We provided additional Co with the Cosecure boluses because the bolus we used to address Se and Cu deficiency was only available with the added Co. We knew from a previous study (Sprinkle et al., 2006) that Co was adequate in range forage on this ranch. Control cattle received the majority of dietary macro and microminerals only from grazed forage with supplemental white iodized salt providing additional Na and I. Control cattle also received modest minerals from the protein supplement that was fed in 2006 and 2008. According to our forage analyses, we would expect control cattle in this study to be deficient for P, Mg, S, Cu (2 yr of 4 yr), Zn, and Se. Unless cattle were able to consistently select a higher quality diet than what was available in the clipped forage we analyzed (Sprinkle et al., 2000), it is to be expected that a loss of production would occur with control cattle which were not provided with any form of mineral supplementation other than white salt.

Table 2 presents the means for the individual forage species analyzed for mineral content over all years of the study. There were numerous differences among forage species but the dominant difference was the increased quantity of some of the minerals which occurred with the half-shrub (shrubby buckwheat). Shrub species have sometimes been shown to have a greater mineral concentration than grasses growing on the same site (Sprinkle et al., 2015).

**Table 2.** Mineral concentrations of forage species across all years^*^

Forage species^†^	Ca, %	P, %	K, %	Mg, %	S, %	Na, %	Zn, ppm	Fe, ppm	Mn, ppm	Cu, ppm	Mo, ppm	Co, ppm	Se,ppm
Ky bluegrass	0.31 ± 0.053	0.21 ± 0.030	0.98 ± 0.156	0.10 ± 0.015	0.16 ± 0.014	0.005 ± 0.007	21.9 ± 3.13	435 ± 225.5	52 ± 11.3	8.8 ± 2.09	0.28 ± 0.167	1.07 ± 0.298	0.034 ± 0.0134
Black grama	0.48 ± 0.066	0.18 ± 0.038	0.84 ± 0197	0.10 ± 0.019	0.11 ± 0.018	0.032 ± 0.009	36.5 ± 3.95	500 ± 285.1	56 ± 14.2	10.7 ± 2.64	0.98 ± 0.211	0.98 ± 0.377	0.062 ± 0.0169
Blue grama	0.35 ± 0.030	0.14 ± 0.017	0.61 ± 0.902	0.11 ± 0.008	0.10 ± 0.008	0.005 ± 0.004	23.3 ± 1.81	462 ± 130.4	33 ± 6.5	7.6 ± 1.21	0.57± 0.096	1.25 ± 0.172	0.057 ± 0.0082
Elk sedge	0.28 ± 0.039	0.17 ± 0.022	1.53 ± 0.114	0.11 ± 0.011	0.10 ± 0.010	0.014 ± 0.005	27.6 ± 2.30	739 ± 165.6	50 ± 8.3	20.2 ± 1.53	0.26 ± 0.122	1.49 ± 0.219	0.050 ± 0.0104
Sand dropseed	0.46 ± 0.066	0.14 ± 0.038	0.67 ± 0.197	0.10 ± 0.019	0.10 ± 0.018	0.008 ± 0.009	31.3 ± 3.95	441 ± 285.1	54 ± 14.2	9.2 ± 2.64	0.95 ± 0.211	0.88 ± 0.377	0.096 ± 0.0169
Shrubby buckwheat	1.10 ± 0.050	0.17 ± 0.028	0.72 ± 0.147	0.30 ± 0.014	0.11 ± 0.013	0.008 ± 0.006	27.5 ± 2.95	1657 ± 213.0	95 ± 10.6	10.2 ± 1.97	0.21 ± 0.213	2.22 ± 0.282	0.125 ± 0.0126
Sideoats grama	0.30 ± 0.039	0.13 ± 0.022	0.55 ± 0.114	0.08 ± 0.011	0.07 ± 0.010	0.009 ± 0.005	27.3 ± 2.30	292 ± 165.6	44 ± 8.3	11.2 ± 1.53	1.00 ± 0.122	0.84 ± 0.219	0.059 ± 0.0099
Slim tridens	0.44 ± 0.066	0.13 ± 0.038	0.67 ± 0.197	0.09 ± 0.019	0.09 ± 0.018	0.006 ± 0.009	26.0 ± 3.95	346 ± 285.1	41 ± 14.2	9.7 ± 2.64	0.90 ± 0.211	0.85 ± 0.377	0.084 ± 0.0169
Spike muhly	0.31 ± 0.030	0.15 ± 0.017	0.54 ± 0.090	0.08 ± 0.008	0.10 ± 0.008	0.005 ± 0.004	24.7 ± 1.81	430 ± 130.4	49 ± 6.5	6.7 ± 1.21	0.26 ± 0.096	1.10 ± 0.172	0.049 ± 0.0081
Squirreltail	0.22 ± 0.039	0.17 ± 0.022	1.12 ± 0.114	0.06 ± 0.011	0.12 ± 0.010	0.004 ± 0.005	17.6 ± 2.30	459 ± 165.6	40 ± 8.3	5.4 ± 1.53	0.37 ± 0.122	0.97 ± 0.219	0.049 ± 0.0099
Threeawn	0.42 ± 0.066	0.14 ± 0.038	0.52 ± 0.197	0.09 ± 0.019	0.09 ± 0.018	0.018 ± 0.009	21.8 ± 3.95	461 ± 285.1	43 ± 14.2	10.2 ± 2.64	1.08 ± 0.211	1.02 ± 0.377	0.096 ± 0.0169
Vine mesquite	0.41 ± 0.050	0.12 ± 0.028	0.67 ± 0.147	0.15 ± 0.014	0.14 ± 0.013	0.006 ± 0.006	24.5 ± 2.95	752 ± 213.0	28 ± 10.6	6.7 ± 1.97	0.60 ± 0.157	1.23 ± 0.282	0.103 ± 0.0126
Western wheatgrass	0.44 ± 0.036	0.15 ± 0.020	1.24 ± 0.106	0.13 ± 0.010	0.13 ± 0.009	0.004 ± 0.005	21.6 ± 2.13	973 ± 153.4	61 ± 7.7	10.6 ± 1.42	0.28 ± 0.113	1.51 ± 0.203	0.074 ± 0.0091
*P*-value	< 0.0001	0.389	< 0.0001	< 0.0001	< 0.0001	0.106	0.0014	0.0003	0.0004	< 0.0001	0.011	0.032	<0.0001
Mid-gestation cow NASEM requirement^‡^	0.15	0.11	0.60	0.12	0.15	0.06 to 0.08	30	50	40	10	–	0.15	0.10
Early lactation cow NASEM requirement^‡^	0.33	0.22	0.70	0.20	0.15	0.10	30	50	40	10	–	0.15	0.10

^*^Least squares means ± SEM on a DM basis; numerous differences existed between forage species which would make this table unwieldy when adding superscripts. Hence, only the treatment *P*-value is shown for each mineral.

^†^Samples obtained from 2006 to 2009 for each species: Kentucky bluegrass (4 from 1 pasture); black grama (4 from 1 pasture); blue grama (12 from 3 pastures); elk sedge (8 from 2 pastures); sand dropseed (4 from 1 pasture); shrubby buckwheat (4 from 1 pasture); sideoats grama (8 from 2 pastures); slim tridens (4 from 1 pasture); spike muhly (12 from 3 pastures); squirreltail (8 from 2 pastures); threeawn (4 from 1 pasture); vine mesquite (4 from 1 pasture); and western wheatgrass (8 from 2 pastures). Samples unable to be analyzed due to limit of instrument quantification included: 2006 (blue grama for Se in 1 pasture); 2007 (shrubby buckwhat for Mo, blue grama for Se for 1 pasture, elk sedge for Se for 1 pasture); 2008 (shrubby buckwheat for Se); 2009 (spike muhly for Se in 1 pasture).

^‡^Based upon Nutrient Requirements of Beef Cattle, National Research Council, 2016. Ca and P requirements are dependent upon cow size, physiological state, and milk production; those shown are for a 480 kg cow with 6 kg peak milk production, 34 kg calf birth weight, and 195 kg adjusted calf weaning weight.

We did not test the forage for iodine and it was assumed that dietary requirements for this trace mineral would be met through feeding the iodized salt. Cattle never had access to *Brassica* forage crops which could increase the requirements for this trace mineral (Lalman and McMurphy, 2017). Additionally, cattle with goiter have never been observed on the ranch.

Beef cattle are not considered to have “nutritional wisdom” (McDowell, 1996), though they will exhibit cravings for some minerals, such as phosphorus, when they are morbidly deficient (McDowell, 1996). Since control cattle were only provided Na and I in this study, they could be assumed to have deficiencies for some minerals during some time periods over the course of this trial. From previous research on this ranch (Sprinkle et al., 2006), we expected the microminerals Cu, Se, and Zn would be deficient. Admittedly, cattle likely consumed a diet slightly better than that indicated by forage sampling (Sprinkle et al., 2000), but we make some projections about the possible intake of Cu and Se (these trace minerals provided to treatment cattle with the Cosecure bolus) by control cattle at two time periods. Allowing for a 20% increase in the dietary concentrations of Cu and Se over the values shown in Figure 1 due to diet selection, forage intake at 2.6% of body weight in mid-lactation and 1.9% of body weight when non-lactating (445 kg in spring and 480 kg in winter), then control cattle could be expected to consume approximately 91% of required dietary Cu and 78% of required dietary Se for the dry year of 2009 (base forage levels for Cu averaged 7.6 ppm and for Se, 0.0649 ppm). In our previous study at this same location (Sprinkle et al., 2006), control and 1X bolused cattle were sampled for liver Cu and whole blood Se. Control cattle in that study had deficient liver Cu when compared to treated cattle, which were adequate (71 ± 6.6 vs. 120 ± 7.5 ppm). Both control and 1X treated cows were marginally deficient in Se in January prior to administering the 6-mo rumen bolus for two of 3 yr (control, 0.088 ± 0.004 and 0.066 ± 0.004; treated, 0.091 ± 0.004 and 0.069 ± 0.003; 2001 and 2002, respectively). With the advent of green grass in May, all cattle had adequate whole blood Se levels (>0.1 ppm, Radostits et al., 1994), though whole blood Se levels were greater (*P* < 0.05) for treated cows. The treatment groups did not differ (*P* > 0.05) in whole blood Se in January for that study.

### Cow Performance

Cows within the 2X bolus treatment had lower body condition in the spring than did either control cows (*P* = 0.045; Table 3) or 1X treated cows (*P* = 0.011; Table 3), though the actual difference was small and likely biologically insignificant. However, a loss of body condition is verified between control and 2X treated H cows by spring cow weights (*P* = 0.037; 2X H = 435 ± 4.9 vs. 448 ± 4.8 kg for control H; Table 3). There was also a tendency (*P* = 0.054; Table 3) for 1X treated H cows to weigh more than 2X H cows in the spring. This trend continued into the fall for H cows, with the 2X cows having lower BCS than 1X cows (*P* = 0.021). Interestingly, an opposite effect appeared to be in place for CGC cows for fall weight, with 2X cows weighing more than 1X cows (*P* = 0.049; Table 3).

**Table 3.** Effects of a long acting trace mineral bolus upon range cow weight and body condition score^*^

	Treatment (TRT)						
Item	*n*	Control	*n*	1X	*n*	2X	TRT *P*
Winter BCS^†^							
All cows and all years	244	4.9 ± 0.04	219	4.9 ± 0.04	227	4.9 ± 0.04	0.948
CGC cows, over all years^‡^	97	4.8 ± 0.06	93	4.9 ± 0.07	89	5.0 ± 0.06	0.342
H cows, over all years^‡^	147	5.0 ± 0.06	126	4.9 ± 0.06	138	4.9 ± 0.06	0.342
Spring BCS^†^							
All cows and all years	218	4.6 ± 0.05^a^	203	4.6 ± 0.04^a^	207	4.5 ± 0.04^b^	0.026
CGC cows, over all years^‡^	92	4.7 ± 0.08^a^	90	4.6 ± 0.06^ab^	82	4.5 ± 0.06^b^	0.516
H cows, over all years^‡^	126	4.5 ± 0.06	113	4.6 ± 0.06	125	4.4 ± 0.06	0.516
Fall BCS^†^							
All cows and all years	313	5.2 ± 0.05	289	5.3 ± 0.05	293	5.2 ± 0.04	0.851
CGC cows, over all years^‡^	132	5.2 ± 0.09	121	5.2 ± 0.07	115	5.3 ± 0.07	0.021
H cows, over all years^‡^	181	5.3 ± 0.06^ab^	168	5.3 ± 0.06^a^	178	5.1 ± 0.06^b^	0.021
Winter wt, kg^†^							
All cows and all years	244	479 ± 4.4	219	480 ± 3.6	228	486 ± 3.6	0.412
CGC cows, over all years^‡^	97	477 ± 7.8	93	480 ± 5.5	89	491 ± 5.4	0.352
H cows, over all years^‡^	147	480 ± 4.6	126	481 ± 4.9	139	480 ± 4.9	0.352
Spring wt, kg^†^							
All cows and all years	199	447 ± 4.5	181	445 ± 3.6	185	443 ± 3.7	0.810
CGC cows, over all years^‡^	83	445 ± 7.7	82	444 ± 5.4	76	451 ± 5.5	0.158
H cows, over all years^‡^	116	448 ± 4.8^a^	99	445 ± 5.1^ab^	109	435 ± 4.9^b^	0.158
Fall wt, kg^†^							
All cows and all years	313	459 ± 4.4	289	457 ± 3.5	293	461 ± 3.5	0.723
CGC cows, over all years^‡^	132	449 ± 7.7^ab^	121	448 ± 5.4^a^	115	463 ± 5.3^b^	0.049
H cows, over all years^‡^	181	468 ± 4.6	168	465 ± 4.8	178	459 ± 4.8	0.049
Change in wt Winter to Spring, kg^†^							
All cows and all years	194	40 ± 2.8	175	41 ± 2.7	181	47 ± 2.7	0.152
CGC cows, over all years^‡^	79	40 ± 4.5	79	41 ± 4.0	75	45 ± 4.1	0.914
H cows, over all years^‡^	115	40 ± 3.5	96	42 ± 3.7	106	49 ± 3.7	0.914
Change in wt Spring to Fall, kg^†^							
All cows and all years	192	16 ± 2.7	177	15 ± 2.6	182	21 ± 2.6	0.298
CGC cows, over all years^‡^	83	10 ± 4.3	79	8 ± 3.8	75	17 ± 3.8	0.652
H cows, over all years^‡^	109	22 ± 3.5	98	22 ± 3.5	107	24 ± 3.5	0.652

^a,b^Means within a row without a common superscript differ (*P* < 0.05).

^*^Cosecure trace mineral boluses had an expected life of approximately 175 d and provided approximately 156 mg/d Cu, 5.9 mg/d Co, and 3.4 mg/d Se. Boluses were provided either at 0, 1X (February or March), or 2X interval (February or March and September or October).

^†^BCS (1–9, 9 = fattest); Winter = February or March; Spring = May or June; Fall = September or October.

^‡^Breeds: CGC = Composite (50% Red Angus, 25% Tarentaise, and 25% Charolais); H = Hereford. Significant main effects for breed were detected for change in wt from spring to fall (*P* = 0.0012) and fall wt (*P* = 0.0371). Year was significant for all dependent variables (*P* < 0.0001).

In the study reported by Sprinkle et al. (2006), cows dosed 1X with Cosecure boluses lost more weight from late gestation to early lactation than did control cows. (*P* = 0.020). The authors hypothesized that this may have been due to increased milk production for treated cows. In this study, we did not find any differences (Table 4) for either breed (*P* = 0.169) or treatment (*P* = 0.951) for milk production at 50 d estimated by weigh-suckle-weigh. We speculate that environmental variation may have overwhelmed any potential treatment differences. Indeed, the only significant difference detected for milk production in this study was for year (*P* < 0.0001) with greater peak milk production following a wet El Niño year in 2008 (7.1 ± 0.30 kg/24 h) compared to 2006 (5.2 ± 0.31 kg/24 h) and 2009 (5.8 ± 0.30 kg/24 h). Tolleson and Schafer (2014) reported greater crude protein and digestibility in cow dietary quality in 2008 than in 2009 (as estimated by near infrared spectroscopy of fecal samples).

**Table 4.** Effects of a long acting trace mineral bolus upon peak 24 h milk production, adjusted weaning wt, yearly calving interval, and calf birth wt*

	Treatment (TRT)						
Item	*n*	Control	*n*	1X	*n*	2X	TRT *P*
24 h milk production, kg^†^							
All cows and all years	54	6.0 ± 0.30	57	6.1 ± 0.29	58	6.1 ± 0.29	0.951
CGC cows, over all years^‡^	26	6.2 ± 0.45	31	6.5 ± 0.40	28	6.2 ± 0.43	0.778
H cows, over all years^‡^	28	5.7 ± 0.42	26	5.7 ± 0.44	30	6.0 ± 0.40	0.778
Adjusted calf weaning wt, kg^†^							
All cows and all years	202	190 ± 2.1^a^	184	194 ± 1.8^ab^	184	196 ± 1.8^b^	0.125
CGC cows, over all years^‡^	88	203 ± 3.5	81	203 ± 2.7	79	207 ± 2.7	0.503
H cows, over all years^‡^	114	178 ± 2.3^a^	103	184 ± 2.4^b^	105	185 ± 2.5^b^	0.503
Actual calf weaning wt, kg^†^							
All cows and all years	202	166 ± 2.5^a^	184	169 ± 2.6^ab^	184	176 ± 2.7^b^	0.040
CGC cows, over all years^‡^	88	178 ± 3.8	81	181 ± 3.9	79	187 ± 4.0	0.992
H cows, over all years^‡^	114	155 ± 3.2^a^	103	158 ± 3.4^ab^	105	164 ± 3.4^b^	0.992
Yearly calving interval, d^†^							
All cows and all years	94	389 ± 2.7^a^	82	382 ± 3.2^ab^	87	378 ± 3.2^b^	0.025
CGC cows, over all years^‡^	52	380 ± 2.9	44	377 ± 4.3	51	372 ± 3.9	0.537
H cows, over all years^‡^	42	399 ± 4.6^a^	38	387 ± 4.7^ab^	36	385 ± 4.9^b^	0.537
Calf birth wt, kg^†^							
All cows and all years	220	33.0 ± 0.28^a^	195	33.9 ± 0.28^b^	194	33.5 ± 0.28^ab^	0.085
CGC cows, over all years^‡^	90	32.8 ± 0.45	85	33.7 ± 0.43	82	33.4 ± 0.43	0.993
H cows, over all years^‡^	130	33.2 ± 0.36	110	34.1 ± 0.38	112	33.7 ± 0.38	0.993

^a,b^Means within a row without a common superscript differ (*P* < 0.05).

^*^Cosecure trace mineral boluses had an expected life of approximately 175 d and provided approximately 156 mg/d Cu, 5.9 mg/d Co, and 3.4 mg/d Se. Boluses were provided either at 0, 1X (February or March), or 2X interval (February or March and September or October).

^†^Milk production determined by weigh-suckle-weigh at 50 d lactation; milk production not determined in 2007 due to a lack of sufficient sample size at peak lactation. Weaning weights adjusted according to Beef Improvement Federation guidelines (BIF, 2018). Actual weaning weights not adjusted for age of calf or dam and actual calf age at weaning was approximately 182 d.

^‡^Breeds: CGC = Composite (50% Red Angus, 25% Tarentaise, and 25% Charolais); H = Hereford. Significant main effects for breed were detected for adjusted weaning wt (*P* < 0.0001) and calving interval (*P* = 0.0002). Year was significant (*P* < 0.0002) for all dependent variables except calf birth wt.

**Figure 2.**
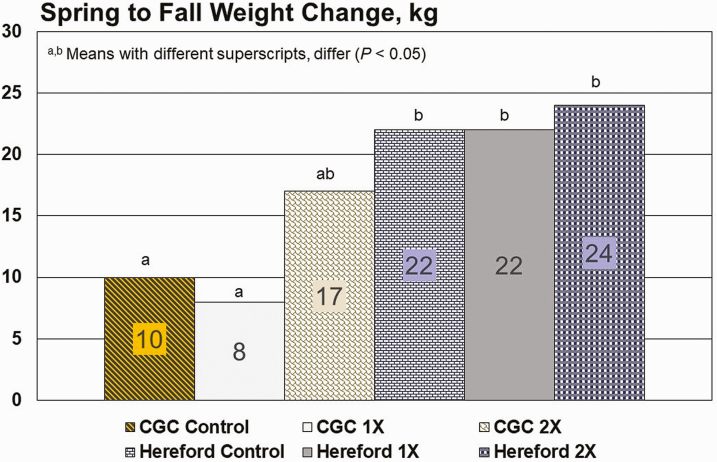
Spring to fall weight change for cattle grazing native range in central Arizona. Cosecure trace mineral boluses had an expected life of approximately 175 d and provided approximately 156 mg/d Cu, 5.9 mg/d Co, and 3.4 mg/d Se. Boluses were provided either at 0, 1X (February or March), or 2X interval (February or March and September or October). Breeds: CGC = Composite (50% Red Angus, 25% Tarentaise, and 25% Charolais); H = Hereford.

**Figure 3.**
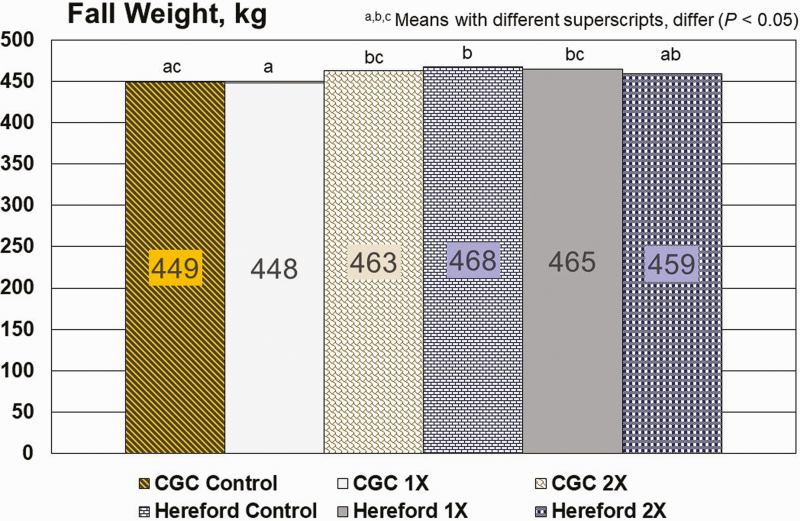
Fall weights for cattle grazing native range in central Arizona. Cosecure trace mineral boluses had an expected life of approximately 175 d and provided approximately 156 mg/d Cu, 5.9 mg/d Co, and 3.4 mg/d Se. Boluses were provided either at 0, 1X (February or March), or 2X interval (February or March and September or October). Breeds: CGC = Composite (50% Red Angus, 25% Tarentaise, and 25% Charolais); H = Hereford.

Year effects were important (*P* < 0.0002) in this study for all variables measured except for calf birth weight (*P* = 0.924). Breed effects were detected for differences in weight change from spring to fall (*P* = 0.0012; Figure 2) and for fall weight (*P* = 0.037; Figure 3). With the exception of the 2X treatment, Hereford cattle had an advantage over CGC cattle for weight gain from spring to fall and for fall weight. This anomaly is likely related to less persistence of the lactation curve for Hereford cattle when compared to crossbred counterparts (Gleddie and Berg, 1968; Casebolt, 1984) with the crossbred cattle having greater persistency. As milk production declined for Hereford cattle, they were able to partition more consumed dietary energy into BW gain. The added minerals made available to CGC 2X cows enabled these cattle to overcome some of the dietary deficits from the native range forage and approach the body weight performance of Hereford cattle from spring to fall.

### Calf Performance Data and Calving Interval

Calf birth weights tended to differ by bolus treatment (*P* = 0.085), being smaller (*P* = 0.027) for control cattle than for 1X treated cattle (Table 4).

Breed effects were detected for differences in adjusted weaning weight (*P* < 0.0001; Table 4), and calving interval (*P* = 0.0002; Table 4). It was expected that breed differences could occur with some of these production characteristics considering we were comparing crossbred vs. purebred cattle. The CGC composite cattle had shorter (*P* = 0.0002) calving interval periods than did H cattle (376 ± 2.2 vs. 390 ± 2.9 d) and weaned heavier (*P* < 0.0001) calves (205 ± 1.8 vs. 182 ± 1.4 kg). There is a preponderance of evidence that supports the assertion that crossbred cows are typically more fertile and wean heavier calves than their purebred counterparts unless breeds are chosen that are a bad fit for the grazing environment (Kress et al., 1990; Gregory et al., 1999; Kress and MacNeil, 1999).

The most striking results from this trial were the effects of increasing trace mineral supply via the boluses upon weaning weight and calving interval. For the overall treatment, the *P*-value differed for calving interval (*P* = 0.025), was significant (*P* = 0.040) for actual weaning weight, but was nonsignificant for adjusted weaning weight (*P* = 0.125). The lack of an overall treatment difference for adjusted weaning weight was due to similarities between control and 1X treatments. However, there was a linear increase for both actual and adjusted weaning weights and linear decrease for calving interval with increased trace mineral supply, being significant at the 2X level for both adjusted weaning weight (*P* = 0.042; Table 4) and calving interval (*P* = 0.009; Table 4) when compared to control cattle. Calves from the 2X treatment weighed 6 kg more (adjusted weaning weight; 10 kg difference for actual weaning weight) than did calves from control cattle and cows on the 2X treatment had yearly calving intervals 11 d shorter (Table 4). For H cattle, cows on the 2X treatment had calving intervals 14 d smaller (*P* = 0.033) that did control cows.

Other research has reported variable results for added Cu, increasing ADG during finishing trials (Ward and Spears, 1997) and decreasing gain for growing dairy heifers (Lopez-Guisa and Satter, 1992). Awadeh et al. (1998) and Gunter et al. (2003) failed to demonstrate any added growth performance for calves nursing Se supplemented cows while Nelson and Miller (1987) reported that weaning weights for calves nursing Se supplemented cows increased by 20 kg.

It appears that any added weight gain for calves nursing cows supplemented with either Cu or Se are dependent upon several factors, chief of which are the dietary Cu or Se concentrations for cows in the study and the presence or absence of any antagonistic trace minerals in the diet such as Mo, Fe, and S. Our pasture concentrations for Cu were adequate to mostly adequate but with a possible negative absorption influence due to high dietary Fe. Villar et al. (2002) reported that positive growth responses appear to occur when dietary Se in the forage base is less than 0.05 ppm DM. The pasture forage Se reported by Gunter et al. (2003) was 0.11 and 0.07 ppm by Awadeh et al. (1998). Our pasture Se concentrations ranged from 0.059 to 0.086 ppm and individual forage species ranged from 0.034 to 0.125 ppm (Table 2).

Strategic supplementation via a long acting trace mineral bolus was successful in decreasing calving interval and increasing calf-weaning weights from cattle grazed in an extensive rangeland environment. At August 2020 calf prices, the value added from increased adjusted weaning weights to cow gross income by the 2X over the control treatment through supplementation would be $19.75 (6 kg = 13.23 lbs. × $1.4925/lb, NM prices, AMS, 2020). Added to this gross profit would be the advantages of a reduced yearly calving interval. For CGC cattle, the added profit for reduced calving interval and 0.825 kg/d calf gain for each day for 8 d would be $21.73 and for H cattle at 0.73 kg/d calf gain over 14 d, the additional profit would be $33.64. The cost of the rumen bolus in the United Kingdom when this study was initiated was $8.70/two-bolus dose in US dollars, or $8.70 for the 1X treatment and $17.40 for the 2X treatment. The current retail price of the boluses in the United Kingdom (Davidsons Farm and Country, Blairgowrie, Scotland, https://www.davidsons.direct/product/cosecure-cattle-20/) for a single two-bolus dose is $9.08 (US dollars, without value added tax or shipping) and this product is not currently available in the United States.
